# The role of sleep deficiency in the relationship between adverse childhood experiences and early adolescent pain outcomes

**DOI:** 10.1002/jcv2.70011

**Published:** 2025-03-23

**Authors:** Thea Senger‐Carpenter, Anao Zhang, Monica Ordway, Sarah A. Stoddard, Terri Voepel‐Lewis

**Affiliations:** ^1^ University of Michigan School of Nursing Ann Arbor Michigan USA; ^2^ University of Michigan School of Social Work Ann Arbor Michigan USA; ^3^ Adolescents and Young Adults Oncology Program at Michigan Medicine Ann Arbor Michigan USA; ^4^ Yale School of Nursing Orange Connecticut USA; ^5^ University of Michigan School of Public Health Ann Arbor Michigan USA; ^6^ Department of Anesthesiology Michigan Medicine Ann Arbor Michigan USA; ^7^ Present address: Department of Family Medicine Michigan State University College of Human Medicine East Lansing MI USA.

**Keywords:** adolescence, adverse childhood experiences, pain, sleep

## Abstract

**Background:**

Sleep deficiency is common among youth exposed to adverse childhood experiences (ACEs) and may contribute towards persistent/recurrent pain (PRP). This study tested the hypotheses that sleep deficiency mediates the effect of ACEs on PRP and moderates the effect of ACEs on PRP exerted through anxiety and depression symptoms.

**Methods:**

We used 4 years of Adolescent Brain Cognitive Development Study® data to test our hypotheses. Annual assessments of sleep duration and quality (from the Sleep Disturbances Scale for Children), pain, anxiety, and depression (from the Child Behavior Checklist) were used to derive our measures. Structural equation modeling and sleep subgroup comparisons estimated effects of early childhood ACEs (measured with parent/youth surveys) on PRP (defined as pain reported for 3 or 4 years) via sleep duration and quality, accounting for effects of anxiety/depression symptoms. Results are presented as standardized adjusted odds ratios with 95% confidence intervals (adj. OR [95% CI]).

**Results:**

Data from 7912 youth were included, nearly one third of whom (*n* = 2527) were classified with PRP by age 12–13. The effect of early childhood ACEs on adolescent PRP was mediated, in part, through insufficient sleep duration (adj. OR 1.01 [95% CI 1.01, 1.02]) and higher sleep quality (adj. OR 0.99 [ 95% CI 0.97, 0.99]). The direct effect of ACEs on PRP was significant only for the subgroup of youth with both insufficient duration and low quality sleep (adj. OR 1.43 [95% CI 1.05, 1.95]). Mediation effects of depression and anxiety were supported across all sleep subgroups with one exception, and sleep did not moderate these associations.

**Conclusion:**

Sleep deficiency may underlie the effect of early ACEs on PRP, though anxiety and depression are likely important to these pathways regardless of sleep. Further investigation into the potentially mechanistic role of sleep deficiency in ACEs/pain associations is warranted.

## Graphical abstract

Sleep deficiency may underlie the negative effects of adverse childhood experiences (ACEs) on adolescent health outcomes, including persistent/recurrent pain (PRP). Using 4 years of Adolescent Brain Cognitive Development Study data, we identified indirect (i.e., mediated) effects of ACEs on early adolescent PRP through interim sleep deficiency, anxiety, and depression symptoms. Findings suggest the potential importance of sleep deficiency to ACEs/pain associations during adolescence.

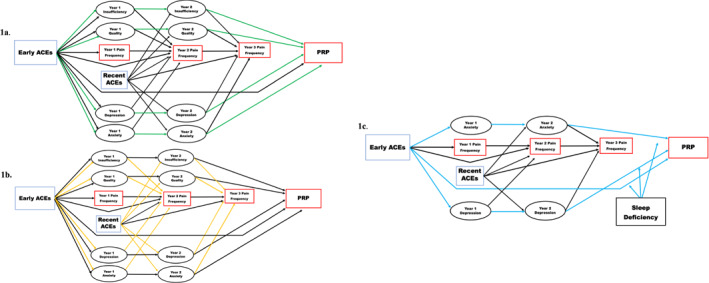

**Key Points**

**What's known?**

- Sleep deficiency frequently emerges during adolescence and may underlie the negative effects of adverse childhood experiences (ACEs) on later health outcomes.
- It is unknown, however, whether or how sleep deficiency affects the association of ACEs with adolescent persistent or recurrent pain (PRP).

**What's new?**

- Using 4 years of data from a national sample of United States adolescents, we identified an effect of childhood ACEs (up to age 9–10 years) on PRP at age 12–13 that was partially mediated through interim sleep deficiency, as well as anxiety and depression symptoms.

**What's relevant?**

- Future work may further investigate the potentially mechanistic role of sleep deficiency in ACEs/PRP associations.

## INTRODUCTION

Adequate sleep is central to health and functioning during adolescence, a developmental stage filled with important milestones (Galvan, 2020; Tarokh et al., 2016). Sleep deficiency, conversely, is associated with many physical and mental health problems, including persistent or recurrent pain (PRP) (Finan et al., 2013; Harrison et al., 2014; Owens et al., 2014). Compared to younger children, adolescents are uniquely vulnerable to sleep deficiency due, in part, to the coincidence of puberty‐related shifts in sleep timing with increasing evening activities and autonomy (Carskadon, 2011; Crowley et al., 2018). Indeed, over half of American adolescents are sleep deficient, with racially and/or socioeconomically marginalized teens disproportionately impacted (Guglielmo et al., 2018; Marco et al., 2011; Wheaton et al., 2018).

Sleep deficiency may be especially impactful for adolescents who have been exposed to adverse childhood experiences (ACEs). Recently, sleep deficiency was proposed as a mechanism through which ACEs negatively impact health across the lifespan (Fuligni et al., 2021). ACE exposure may trigger over‐activation of stress responses (i.e., “toxic stress”), leading to multisystemic biobehavioral dysregulation (e.g., alterations in neural structure and functioning, wide‐spread inflammation) (Fuligni et al., 2021; Shonkoff et al., 2012). The sequelae of such dysregulation (e.g., hypervigilance, anxiety, depression, impaired emotion regulation, cognitive processing, and social functioning) may increase the risk for sleep deficiency and other health problems, while simultaneously being worsened by sleep deficiency (Fuligni et al., 2021).

Critically, the same patterns of dysregulation which may lead to (and be exacerbated by) sleep deficiency are thought to underlie the association of ACEs with PRP (Nelson et al., 2017). While few studies have examined pathways from ACEs to PRP via sleep deficiency, recent evidence suggests that aspects of sleep deficiency partially explain relationships between cumulative ACEs and chronic pain, as well as relationships between post‐traumatic stress symptoms and pain outcomes among adolescents (Noel et al., 2018; Pavlova et al., 2020; Roman‐Juan et al., 2023). These findings suggest a potential role for sleep deficiency as a mechanistic link between ACEs and other health outcomes, including PRP.

Anxiety and depression symptoms are likely important to the associations between ACEs, sleep deficiency, and PRP. Core aspects of anxiety and depression symptoms such as rumination, catastrophizing, negative affect, and withdrawal may interact with other youth factors including ACE exposure to contribute towards pain persistence (Vinall et al., 2016). Indeed, emerging data support hypothesized pathways from ACEs to anxiety/depression symptoms, which, in turn, increase the risk for pain persistency (Hammond et al., 2019; Senger‐Carpenter et al., 2024). Anxiety and depression symptoms are also closely related to sleep deficiency, particularly during early adolescence when such symptoms often emerge or escalate (McMakin & Alfano, 2015; Shimizu et al., 2021). Specifically, evidence suggests that adolescent sleep deficiency may predict and/or sustain anxiety and depression symptoms, while treating sleep problems with evidence‐supported interventions (e.g., cognitive behavioral therapy) may improve adolescent mental health (Blake & Allen, 2020; Goldstone et al., 2020).

Notably, burgeoning data also suggest that sleep deficiency amplifies the effect of ACEs on adolescent mental health symptoms and behaviors, but that healthy sleep is attenuative (Richardson et al., 2019; Tu et al., 2015; Venta & Alfano, 2021). For instance, the association of ACEs with anxiety symptoms was stronger among Spanish adolescents with chronic pain who had high levels of sleep disturbances, compared to those with low levels (Roman‐Juan et al., 2023). Despite these associations, it remains unclear whether and how adolescent sleep deficiency, anxiety, and depression symptoms interact to impact the risk of PRP among youth exposed to ACEs.

**FIGURE 1:**
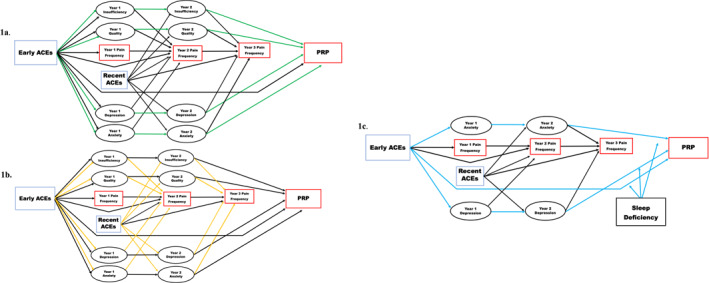
Conceptual Models Hypothesis 1 (A), Hypothesis 2 (B) and Hypothesis 3 (C). Ovals represent latent variables, squares observed variables. Green, gold, and blue lines represent mediated relationships under study in each hypothesis. Error terms, covariates, and cross‐sectional associations are not depicted for readability. ACEs, adverse childhood experiences; PRP, persistent/recurrent pain.

Understanding how sleep deficiency impacts the effects of ACEs on PRP may guide future prevention and intervention efforts to target sleep deficiency alone, or in combination with other factors (e.g., anxiety and depression symptoms). The primary purpose of this study was, therefore, to evaluate sleep deficiency as an underlying mechanism of the relationship between ACEs and pain outcomes across early adolescence. Second, we examined whether sleep deficiency impacted the degree to which ACEs affected PRP directly, or indirectly via anxiety and depression symptoms. To that end, we hypothesized that sleep deficiency would (1) mediate the effect of early childhood ACEs (by age 9–10 years) on early adolescent PRP (age 12–13 years); (2) mediate the effects of early and recent ACEs (at age 10–11 years) on subsequent pain frequency measured at ages 10–11 and 11–12 years; and (3) strengthen (i.e., moderate) the direct effect of ACEs on PRP, as well as the indirect effects of ACEs on PRP via interim anxiety and depression symptoms (Figure 1A–C).

## METHODS AND MATERIALS

### Data and participants

**FIGURE 2:**
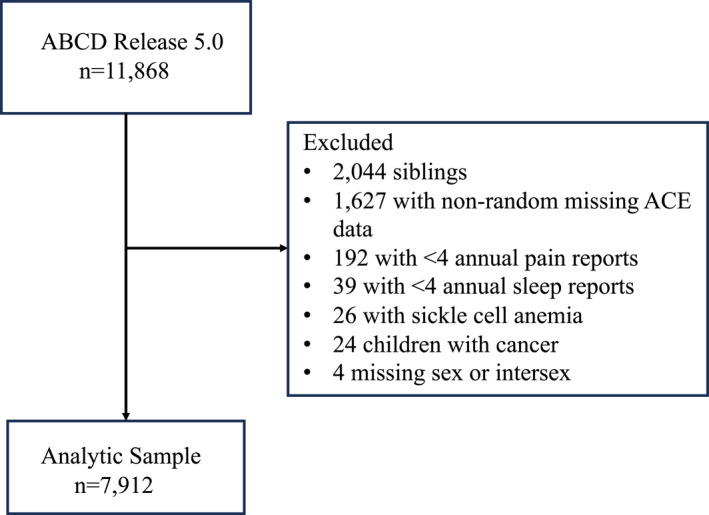
STrengthening the reporting of OBservational studies in Epidemiology (STROBE) flow diagram showing participant selection for this study. ABCD, adolescent brain cognitive development; ACE, adverse childhood experiences.

We conducted secondary analyses of Adolescent Brain Cognitive Development Study data collected from 2016 to 2022 (ABCD® Release 5.0). ABCD® is an ongoing longitudinal study of child development that recruited and consented 11,880 9–10 year olds from 21 sites across the United States (Compton et al., 2019; Garavan et al., 2018). ABCD® participants and one caregiver complete annual assessments of symptoms and behaviors and, as of year 3, attrition has been minimal (0.01% withdrew; 4% late/missing visits) (Feldstein Ewing et al., 2022). Data are available from 11,868 youth at baseline, 11,220 at year 1 (age 10–11 years), 10, 973 at year 2 (age 11–12 years) and 10, 336 at year 3 (age 12–13 years) (ABCD® Study, 2024). For these analyses, we included baseline through year 3 data from one child per family, selected at random to eliminate clustering effects (Marshall et al., 2023). Youth who reported having sickle cell anemia or cancer at baseline, who had non‐random missing ACE data, or who completed fewer than four annual pain and/or sleep assessments were excluded (see Figure 2). This study was approved by the University of Michigan Institutional Review Board (HUM00225649) and a data use agreement was established with the National Institute of Mental Health (DAR#15008).

### Measures

#### Outcomes

##### Annual pain frequency

Annual pain frequency was measured using three items from the parent‐reported Child Behavior Checklist (CBCL) which elicit the presence and frequency of youth's headache, abdominal, and generalized body pain complaints over the last 6 months (Achenbach & Rescorla, 2001). Each item is scored 0 (never), 1 (sometimes) or 2 (often), with summed scores ranging from 0 (no pain complaints of any type) to 6 (child complains of pain often, of all three types [e.g., headache, abdominal, and body]). The construct validity of these items has been supported for youth with various pain conditions (e.g., headache and recurrent abdominal pain; Galli et al., 2007). Further, rates of headache, abdominal, and generalized pain have been used to estimate the overall prevalence of adolescent PRP (Chambers et al., 2024; King et al., 2011).

##### Persistent/recurrent pain (PRP)

Youth who complained of pain at three or more of the 4 years (i.e., repeated complaints of one or more pain types across years) were considered to have PRP. This approach is similar to assessments of adolescent pain recurrence (Campo et al., 2002; Senger‐Carpenter et al., 2024; van Gessel et al., 2011), pain and co‐occurring psychological and non‐painful somatic symptom trajectories (Voepel‐Lewis et al., 2023) and new‐onset multisite pain (Kaplan et al., 2023) in previous studies.

#### Exposure

##### Adverse childhood experiences (ACEs)

Early (by age 9–10 years, reported at baseline) and recent (at age 10–11 years, reported at year 1) ACE exposures were measured using items from multiple youth and parent‐completed surveys and scales, similar to other ABCD® research (Nagata et al., 2023; Stinson et al., 2021; see Table S1). Items were selected to approximate the Pediatric ACEs and Related Life Events Screener (Koita et al., 2018) to the extent permitted by data availability, as ACE surveys are administered annually or every other year in the ABCD® protocol. Early ACEs therefore included physical, emotional, and sexual abuse; physical and emotional neglect; parents' mental health and substance use problems; parental divorce or separation; familial financial adversity; witnessing interpersonal violence; exposure to community violence; and peer victimization. Recent ACEs included physical and emotional neglect, family mental health and substance use problems, parental divorce or separation, parental imprisonment, familial financial adversity, peer victimization, and perceived discrimination. Each endorsed experience was coded as 1 and summed to yield scores of 0–12 (early) or 0–9 (recent). ACE exposures reported at years 2 and 3 were included only as covariates to account for any effects on the associations of interest (see Figure S1).

#### Mediators and moderators

##### Sleep deficiency

Aspects of sleep deficiency included nightly sleep duration and quality. These were derived using items from the annual parent‐reported Sleep Disturbance Scale for Children (SDSC). The SDSC measures past 6‐month sleep behaviors and disturbances with established reliability (Cronbach's alpha 0.71–0.79) and diagnostic validity (Area Under the Curve analysis = 0.91) for adolescents (Bruni et al., 1996).

##### Sleep duration

Average nightly sleep duration was measured annually using the item “How many hours of sleep does your child get on most nights?” as per prior research (Goldstone et al., 2020). Response options included 9–11 h, 8–9 h, 7–8 h, 5–7 h or less than 5 h. Participants were classified as having an insufficient duration if they obtained less sleep than recommended by the American Academy of Sleep Medicine for their age group (e.g., 9–12 h for ages 6–12 years; 8–10 h for ages 13–18 years) (Paruthi et al., 2016).

##### Sleep quality

Sleep quality was measured using the sum of four items assessing sleep initiation (“the child has difficulty getting to sleep at night”), sleep latency (“how long after going to bed does your child usually take to fall asleep?”), and wakefulness after sleep onset (“the child wakes up more than twice per night”, “after waking up in the night the child has difficulty falling back asleep”). Items were rated 0 (never) to 4 (always/daily) and summed, such that greater scores (range 0–16; reverse coded) indicated higher sleep quality. The Cronbach's alpha ranged from 0.70 (baseline) to 0.74 (year 3).

##### Sleep subgroups

Participants were categorized into subgroups based on duration and quality to allow for a multidimensional consideration of sleep deficiency (Meltzer et al., 2021). Insufficient duration was defined as not meeting age‐appropriate sleep duration recommendations for three or more of the 4 years. Sleep quality was dichotomized based on having sleep quality scores in the lower sample quartile across years (low quality) or combined higher quartiles (high sleep quality). Sleep subgroups were coded as Sufficient/High Quality, Insufficient/High Quality, Sufficient/Low Quality, and Insufficient/Low Quality, with this latter group exhibiting the most sleep deficiency.

##### Anxiety symptoms

Anxiety symptoms over the past 6 months were measured annually by the 13‐item parent‐reported CBCL Anxious/Depressed subscale (score range 0–26). The Anxious/Depressed subscale has demonstrated internal consistency (Cronbach's alpha 0.82), test‐retest reliability (Pearson's *r* = 0.82), criterion and construct validity among adolescents (Achenbach & Rescorla, 2001; Aschenbrand et al., 2005).

##### Depression symptoms

Past 6‐month depression symptoms were measured annually by the 8‐item parent‐reported CBCL Withdrawn/Depressed subscale (score range 0–16). The internal consistency (Cronbach's alpha = 0.89), test‐retest reliability (Pearson's *r* = 0.80), construct and criterion validity for this subscale are supported for adolescents (Achenbach & Rescorla, 2001; Aschenbrand et al., 2005).

#### Covariates

##### Demographics

Youths' biological sex at birth, race and Hispanic ethnicity, highest level of parental education, and household income to needs ratio (HINR) were derived from a parent‐reported baseline demographics survey (Barch et al., 2018). Race was categorized as Black, Multiracial, White, or Other. The highest level of completed education reported by the parent accompanying the child to their baseline assessment was categorized as a high school/GED degree or less, some college or an associate's degree, bachelor's/professional degree, or post‐graduate degree. HINR was calculated by dividing youths' baseline annual household income by the 2016 federal poverty line for their family size, with higher values representing greater wealth (Gonzalez et al., 2020).

##### Medical comorbidities

At baseline, parents reported on 10 chronic conditions (e.g., seizures, asthma, diabetes) for which the child had ever seen a provider. To account for possible effects on sleep deficiency, PRP, and anxiety/depression symptoms, endorsed conditions were summed to create a baseline comorbidity index, which was then dichotomized as 0–1 versus ≥2 conditions.

##### Acute medical conditions

Similarly, 16 acute conditions (e.g., injuries, burns, febrile illnesses) were summed annually and dichotomized as 0 versus ≥1.

## ANALYSES

We used Stata SE version 18.0 to code and describe the data. Group characteristics are presented as frequency (%) or mean ± standard deviation. ACE reports could not be considered missing completely at random due to their sensitive nature and high levels of missingness in the sample (up to 16%). Therefore, we excluded participants missing ACE data present for most of the sample (i.e., those with non‐random missing ACE data, *n* = 1627). Remaining levels of missingness (<7% across variables) were addressed using full information maximum likelihood estimation.

### Measurement models

We used MPlus version 8.0 to conduct structural equation modeling (SEM). First, annual anxiety and depression symptoms were modeled as latent variables using all items (i.e., indicators) from the CBCL anxiety and depression subscales. Modeling symptoms as latent variables allows for the correlation of error terms to account for measurement error, a major strength of SEM (Ullman & Bentler, 2012). Model fit and invariance were evaluated using the root mean squared error of approximation (RMSEA) and standardized root mean squared residual (SRMR), both considered acceptable at <0.06–0.08, as well as the comparative fit index (CFI) and Tucker‐Lewis Index (TLI; acceptable at ≥0.90–0.95) (Kline, 2015). A significant chi squared statistic was anticipated given the size of the ABCD® cohort.

### Structural models

Next, we constructed two autoregressive cross‐lagged panel models. The first estimated the direct and indirect (i.e., mediated) effects of (1) early ACEs on PRP and of (2) early and recent ACEs on subsequent pain frequency scores via interim sleep insufficiency and quality, anxiety, and depression symptoms (see Figure 1A,B). The second model estimated the direct and indirect effects of early ACEs on PRP via interim anxiety and depression symptoms only. This model was used to conduct multigroup comparison analyses examining for differences in the direct and indirect effects of early ACEs on PRP via interim anxiety and depression symptoms by sleep subgroup (i.e., moderated mediation) (Figure 1C).

Each model included relevant covariates (i.e., sex, race, ethnicity, comorbidity indices, parental education, and HINR). Further, each accounted for multiple concurrent and longitudinal pathways which could impact the hypothesized associations. These included pathways from baseline to years 1, 2, and 3 pain frequency scores, as well as pathways from baseline to years 1, 2, and 3 sleep insufficiency and quality, anxiety and depression symptoms. The weighted least squares mean and variance adjusted estimator, theta parameterization, and probit link were used to accommodate the inclusion of categorical variables in the models. Path model fit was evaluated using the RMSEA, SRMR, CLI, and TFI. Standardized beta coefficients and odds ratios (OR) are reported with 95% confidence intervals.

### Hypotheses tests

To address hypotheses 1 and 2, we used the joint significance approach to mediation to test for indirect effects of early ACEs on PRP via interim sleep insufficiency and/or quality, as well as for indirect effects of early and recent ACEs on subsequent pain frequency scores via interim sleep insufficiency/quality. Strengths of the joint significance approach include the ability to investigate multiple mediation relationships in a single analysis, including multiple exposures, mediators, and/or outcomes (Gunzler et al., 2013). Direct or unmediated effects (e.g., early ACEs→PRP), indirect or mediated effects (e.g., early ACEs→sleep quality→PRP), and total effects (the sum of these two) were obtained using the *model indirect* command (Gunzler et al., 2013). Hypothesis 1 was considered supported if there were significant (*p* < 0.05) indirect effects of early ACEs on PRP via interim sleep insufficiency and/or quality reported at both years 1 and 2. Hypothesis 2 was considered supported by significant (*p* < 0.05) indirect effects of (a) early ACEs on subsequent pain frequency reported at year 2 via interim sleep insufficiency and/or quality reported at year 1, and of (b) recent ACEs on subsequent pain frequency at year 3 via interim sleep insufficiency and/or quality reported at year 2.

For hypothesis 3, we conducted multigroup comparison analyses to test whether (1) sleep deficiency moderated the direct effect of early ACEs on PRP; and (2) whether sleep deficiency moderated the indirect effects of early ACEs on PRP via interim anxiety and depression symptoms (i.e., moderated mediation). Moderated mediation occurs when the strength of an indirect effect depends on another variable (Kline, 2015). Within the SEM framework, moderation and moderated mediation can be examined by categorizing the sample into subgroups based on the hypothesized moderator and estimating the direct and indirect effects within each (Edwards & Lambert, 2007; Kline, 2015). This approach facilitates an examination of both direct and indirect effects for moderation, while holding all other relationships constant (Kline, 2015). While subgrouping can lower statistical power (Edwards & Lambert, 2007), the size of the ABCD® cohort easily accommodates this approach. The significance of observed differences in direct or indirect effects between groups can be tested using the Wald Test of Parameter Constraints for direct pathways, and the Mplus *model constraint* command for indirect pathways.

Multigroup comparison analyses were conducted using the four sleep subgroups: Sufficient/High Quality, Insufficient/High Quality, Sufficient/Low Quality, and Insufficient/Low Quality. The second cross‐lagged panel model estimating the effects of early ACEs on PRP through anxiety and depression symptoms was estimated for each subgroup (see Figure 1C). Unconstrained models (i.e., models for which the value of parameters were allowed to vary) were compared to constrained models with forced equality of direct or indirect effects. Hypothesis 3 was considered supported by significant differences (*p* < 0.05) between the four subgroups in the direct effects of early ACEs on PRP, and in the indirect effects of early ACEs on PRP through interim anxiety and depression symptoms reported at years 1 and 2.

## RESULTS

The analytic sample included 7912 youth (see Figure 2), close to one third of whom (*n* = 2,527, 31.9%) were classified with PRP. Youth with and without PRP are described in Table 1, and ACE exposures in these groups are described in Table 2. The Sufficient/High Quality sleep subgroup was the largest (*n* = 3459; 43.7%), while 2211 youths (27.9%) had Insufficient/High Quality, 1048 (13.3%) had Sufficient/Low Quality, and 1194 (15.1%) had Insufficient/Low Quality sleep. The sleep subgroups are fully described in Table 3.

**TABLE 1:** Description of PRP and no‐PRP groups (*n* = 7912).

	PRP (*n* = 2527)	No PRP (*n* = 5385)	*p*‐value
Child and family characteristics			
Female sex	1293 (51.2)	2436 (45.2)	<0.001
Hispanic ethnicity	469 (18.8)	1145 (21.5)	0.006
Race			<0.001
White	1748 (69.2)	3453 (64.1)	–
Black	276 (10.9)	786 (14.6)	–
Multiracial	348 (13.8)	642 (11.9)	–
Other	155 (6.1)	497 (9.2)	–
Baseline comorbidity index ≥2 (vs. 0–1)	921 (36.5)	1270 (23.6)	<0.001
Baseline acute conditions ≥1 (vs. 0)	1498 (59.3)	2671 (49.6)	<0.001
Year 1 acute conditions	663 (26.2)	1012 (18.8)	<0.001
Year 2 acute conditions	452 (17.9)	672 (12.5)	<0.001
Year 3 acute conditions	250 (9.9)	388 (7.2)	<0.001
Parent education			<0.001
≤High school	308 (12.2)	850 (15.8)	–
Some college/associate's degree	812 (32.2)	1417 (26.4)	–
Bachelor's degree	761 (30.1)	1582 (29.4)	–
Post‐graduate degree	645 (25.5)	1528 (28.4)	–
Household income to needs ratio	3.8 ± 2.5	3.9 ± 2.5	0.279
Baseline pain frequency (0–6)	1.6 ± 1.1	0.30 ± 0.65	<0.001
Year 1 pain frequency	1.6 ± 1.1	0.27 ± 0.62	<0.001
Year 2 pain frequency	1.6 ± 1.1	0.24 ± 0.57	<0.001
Year 3 pain frequency	1.5 ± 1.0	0.27 ± 0.62	<0.001
Sleep duration and quality			
Baseline insufficiency	1304 (51.6)	2680 (49.8)	0.128
Year 1 insufficiency	1572 (62.2)	3168 (58.8)	0.004
Year 2 insufficiency	1792 (70.9)	3589 (66.7)	<0.001
Year 3 insufficiency	759 (30.0)	1328 (24.7)	<0.001
Baseline sleep quality (0–16)	13.9 ± 2.2	13.0 ± 2.6	<0.001
Year 1 sleep quality	13.7 ± 2.3	12.8 ± 2.7	<0.001
Year 2 sleep quality	13.7 ± 2.3	12.6 ± 2.9	<0.001
Year 3 sleep quality	13.5 ± 2.5	12.3 ± 2.9	<0.001
Sleep subgroups			
Sufficient duration and high quality	931 (36.8)	2528 (47.0)	<0.001
Insufficient duration and high quality	608 (24.1)	1603 (29.8)	<0.001
Sufficient duration and low quality	428 (16.9)	620 (11.5)	<0.001
Insufficient duration and low quality	560 (22.2)	634 (11.8)	<0.001
Mental health symptoms			
Baseline anxiety (0–26)	3.6 ± 3.6	2.1 ± 2.6	<0.001
Year 1 anxiety	3.7 ± 3.5	2.1 ± 2.7	<0.001
Year 2 anxiety	3.5 ± 3.5	1.8 ± 2.5	<0.001
Year 3 anxiety	3.5 ± 3.7	1.9 ± 2.6	<0.001
Baseline depression (0–16)	1.4 ± 2.0	0.81 ± 1.5	<0.001
Year 1 depression	1.6 ± 2.1	0.91 ± 1.5	<0.001
Year 2 depression	1.8 ± 2.4	1.0 ± 1.7	<0.001
Year 3 depression	2.1 ± 2.6	1.2 ± 1.9	<0.001

*Note*: Data presented as *n* (% calculated from those with complete data for the variable) or mean ± standard deviation as appropriate.

Abbreviation: PRP, persistent or recurrent pain.

*p*‐values from chi square or *t*‐test.

**TABLE 2:** ACE exposures in the PRP and no PRP Groups (*n* = 7912).

	PRP (*n* = 2527)	No PRP (*n* = 5385)	*p*‐value
Early ACE exposures			
Physical abuse	24 (1.0)	41 (0.80)	0.381
Sexual abuse	64 (2.6)	72 (1.4)	<0.001
Emotional abuse	35 (1.4)	38 (0.72)	0.003
Physical neglect	98 (3.9)	297 (5.5)	0.002
Emotional neglect	10 (0.40)	28 (0.52)	0.457
Parent mental health problem	1467 (61.8)	2257 (45.2)	<0.001
Parent substance use problem	588 (23.9)	866 (16.6)	<0.001
Witnessing interpersonal violence	244 (9.9)	308 (5.9)	<0.001
Parent divorce or separation	356 (14.1)	632 (11.8)	0.003
Family financial adversity	617 (24.5)	944 (17.6)	<0.001
Community violence	17 (0.70)	43 (0.82)	0.554
Peer victimization	506 (20.0)	683 (12.7)	<0.001
Average early exposures (0–12)	1.6 ± 1.4 (0–7)	1.2 ± 1.3 (0–9)	<0.001
Recent ACE exposure			
Physical neglect	33 (1.3)	142 (2.6)	<0.001
Emotional neglect	5 (0.20)	12 (0.22)	0.824
Family mental health problem	293 (11.6)	453 (8.4)	<0.001
Family substance use problem	321 (12.7)	584 (10.9)	0.015
Parent imprisonment	203 (8.0)	341 (6.3)	0.005
Parent divorce or separation	83 (3.8)	180 (3.7)	0.834
Family financial adversity	608 (24.2)	922 (17.2)	<0.001
Peer victimization	543 (21.5)	769 (14.3)	<0.001
Experiencing discrimination	264 (12.1)	447 (9.6)	0.001
Average recent exposures (0–9)	0.85 ± 1.1 (0–6)	0.66 ± 0.93 (0–6)	<0.001

*Note*: Data presented as *n* (% calculated from those with complete data for the variable), mean ± standard deviation or (range) as appropriate.

Abbreviations: ACE, adverse childhood experience; PRP, persistent or recurrent pain.

*p*‐values from chi squared or *t*‐tests.

**TABLE 3:** Description of sleep subgroups (*n* = 7912).

	Sufficient duration/high quality (*n* = 3459)	Insufficient duration/high quality (*n* = 2211)	Sufficient duration/low quality (*n* = 1048)	Insufficient duration/low quality (*n* = 1194)	*p*‐value
Child and family characteristics					
Female sex	1584 (45.8)	1029 (46.5)	533 (50.9)	583 (48.8)	0.018
Hispanic ethnicity	612 (17.9)	638 (29.3)	130 (12.5)	234 (19.9)	<0.001
Race					<0.001
White	2537 (73.4)	1172 (53.1)	791 (75.6)	701 (58.7)	–
Black	252 (7.3)	513 (23.2)	66 (6.3)	231 (19.4)	–
Multiracial	405 (11.7)	270 (12.2)	140 (13.4)	175 (14.7)	–
Other	262 (7.6)	253 (11.4)	50 (4.8)	87 (7.3)	
Baseline comorbidity index ≥2 (vs. 0–1)	874 (25.3)	566 (25.6)	336 (32.1)	415 (34.8)	<0.001
Baseline acute conditions ≥1 (vs. 0)	1844 (53.3)	1046 (47.3)	597 (57.0)	682 (57.1)	<0.001
Year 1 acute conditions	688 (19.9)	428 (19.4)	244 (23.3)	315 (26.4)	0.000
Year 2 acute conditions	492 (14.2)	271 (12.3)	179 (17.1)	182 (15.2)	0.002
Year 3 acute conditions	271 (7.8)	146 (6.6)	104 (9.9)	117 (9.8)	0.001
Parent education					<0.001
≤ High school	341 (9.9)	522 (23.7)	87 (8.3)	208 (17.5)	–
Some College/Associate's degree	797 (23.1)	760 (34.4)	231 (22.0)	441 (37.0)	–
Bachelor's degree	1187 (34.4)	513 (23.2)	348 (33.2)	295 (24.8)	–
Post‐graduate degree	1131 (32.7)	412 (18.7)	382 (36.5)	248 (20.8)	–
Household income to needs ratio	4.4 ± 2.5	3.1 ± 2.4	4.4 ± 2.4	3.3 ± 2.4	<0.001
Adverse childhood experiences (ACEs)					
Early ACEs (0–12)	1.1 ± 1.2	1.4 ± 1.4	1.4 ± 1.3	1.8 ± 1.5	<0.001
Interim ACEs (0–9)	0.55 ± 0.85	0.84 ± 1.0	0.68 ± 0.95	1.1 ± 1.2	<0.001
Pain symptoms					
Baseline pain frequency (0–6)	0.58 ± 0.89	0.61 ± 0.91	0.90 ± 1.1	1.0 ± 1.2	<0.001
Year 1 pain frequency	0.58 ± 0.88	0.59 ± 0.91	0.92 ± 1.1	1.1 ± 1.2	<0.001
Year 2 pain frequency	0.57 ± 0.90	0.56 ± 0.93	0.90 ± 1.1	1.0 ± 1.2	<0.001
Year 3 pain frequency	0.55 ± 0.85	0.57 ± 0.90	0.85 ± 1.1	1.0 ± 1.2	<0.001
Persistent/Recurrent pain (PRP)	931 (26.9)	608 (27.5)	428 (40.8)	560 (46.9)	<0.001
Mental health symptoms					
Baseline anxiety (0–26)	2.1 ± 2.5	2.1 ± 2.7	3.4 ± 3.5	4.0 ± 3.9	<0.001
Year 1 anxiety	2.2 ± 2.6	2.0 ± 2.6	3.6 ± 3.6	4.0 ± 3.8	<0.001
Year 2 anxiety	2.0 ± 2.6	1.8 ± 2.7	3.3 ± 3.4	3.5 ± 3.6	<0.001
Year 3 anxiety	2.0 ± 2.6	1.8 ± 2.6	3.5 ± 3.7	3.7 ± 3.8	<0.001
Baseline depression (0–16)	0.74 ± 1.3	0.89 ± 1.6	1.3 ± 1.8	1.7 ± 2.2	<0.001
Year 1 depression	0.85 ± 1.4	1.0 ± 1.7	1.4 ± 1.9	2.0 ± 2.3	<0.001
Year 2 depression	0.96 ± 1.6	1.1 ± 1.9	1.6 ± 2.1	2.1 ± 2.5	<0.001
Year 3 depression	1.2 ± 1.9	1.2 ± 1.9	2.1 ± 2.5	2.5 ± 2.8	<0.001

*Note*: Data presented as *n* (% calculated from those with complete data for the variable) or mean ± standard deviation as appropriate.

Abbreviation: ACE, adverse childhood experience.

*p*‐values from chi squared or analysis of variance (ANOVA).

### Hypothesis 1

Our first hypothesis was fully supported by the first cross‐lagged panel model (model fit RMSEA 0.02; SRMR 0.05; CFI 0.97; TLI 0.96). Early ACEs increased the likelihood of having PRP through interim sleep insufficiency at years 1 and 2 (adj. standardized OR 1.01 [1.01, 1.02]), suggesting that part of the effect of early ACEs on PRP was exerted through sustained sleep deficiency from ages 10–11 to 11–12 years (see Table 4). Conversely, there was a negative indirect effect of early ACEs on PRP through interim sleep quality at years 1 and 2 (adj. standardized OR 0.99 [0.97, 0.99]). That is, higher sleep quality across early adolescence attenuated part of the effect of early ACEs on PRP.

**TABLE 4:** Effects of early and recent ACEs on PRP and subsequent pain frequency.^a^

	Unstandardized adj. OR [95% CI], *p*‐value	Standardized adj. OR [95% CI], *p*‐value
Hypothesis 1		
Total effect of early ACEs on PRP	1.10 [1.05, 1.16], <0.001	1.32 [1.18, 1.47], <0.001
Direct effect of early ACEs on PRP	1.19 [1.11, 1.27], <0.001	1.64 [1.24, 2.16], <0.001
Indirect effect through interim^b^ sleep quality	0.99 [0.98, 0.99], 0.027	0.99 [0.97, 0.99], 0.027
Indirect effect through interim sleep insufficiency	1.01 [1.00, 1.01], <0.001	1.01 [1.01, 1.02], <0.001

	Unstandardized adj. *þ* [95% CI], *p*‐value	Standardized adj. *þ* [95% CI], *p*‐value
Hypothesis 2a		
Total effect of early ACEs on subsequent pain frequency	0.23 [0.17, 0.28], <0.001	0.30 [0.23, 0.37], <0.001
Direct effect of early ACEs on subsequent pain frequency	−0.04 [−0.10, 0.01], 0.121	−0.06 [−0.13, 0.01], 0.121
Indirect effect through interim sleep quality	0.00 [−0.01, 0.01], 0.461	0.01 [−0.01, 0.02], 0.461
Indirect effect through interim sleep insufficiency	0.01 [0.00, 0.01] 0.031	0.01 [0.00, 0.01], 0.030
Hypothesis 2b		
Total effect of recent ACEs on subsequent pain frequency	0.03 [0.01, 0.06], 0.006	0.03 [0.01, 0.06], 0.006
Direct effect of recent ACEs on subsequent pain frequency	0.03 [0.01, 0.06], 0.013	0.03 [0.01, 0.06], 0.010
Indirect effect through interim sleep quality	0.00 [−0.00, 0.01], 0.340	0.00 [−0.00, 0.00], 0.340
Indirect effect through interim sleep insufficiency	0.00 [−0.00, 0.00] 0.322	0.00 [−0.00, 0.00], 0.322
Model fit: RMSEA 0.02; SRMR 0.05; CFI 0.97; TLI 0.96; Chi^2^ 8096.6, *p* < 0.001		

*Note*: Coefficients are adjusted for relevant covariates and additional associations.

Abbreviations: ACEs, adverse childhood experiences; CI, confidence interval; OR, odds ratio; PRP, persistent or recurrent pain.

^a^ Subsequent pain frequency measured at year 2 (hypothesis 2a) and year 3 (hypothesis 2b).

^b^ Interim sleep quality and insufficiency measured at years 1 and 2 (hypothesis 1), year 1 (hypothesis 2a), or year 2 (hypothesis 2b).

### Hypothesis 2

The second hypothesis was partially supported by an indirect effect of early ACEs on subsequent pain frequency (year 2) via interim sleep insufficiency (year 1) (adj. standardized β 0.01 [0.00, 0.01]) but not sleep quality (see Table 4). There were no indirect effects of recent ACEs on subsequent pain frequency (year 3) via sleep insufficiency or quality. Thus, in our sample, part of the effect of early ACEs on subsequent pain frequency depended upon sleep insufficiency but not quality, accounting for additional indirect effects through interim anxiety and depression symptoms.

### Hypothesis 3

Our third hypothesis was refuted, as the second cross‐lagged panel model did not support a moderation effect of sleep deficiency on the direct effect of early ACEs on PRP, nor on the indirect effects of early ACEs on PRP through anxiety and depression symptoms (see Tables 5 and 6). However, there were observable differences in the strength and significance of associations between the sleep subgroups. For instance, the direct effect of early ACEs on PRP was significant only in the worst sleep subgroup (adj. standardized OR 1.43 [1.05, 1.95] see Table 5), suggesting that the effect of early ACEs on PRP was fully explained by anxiety and depression in the other sleep groups. Indeed, early ACEs indirectly effected PRP via depression symptoms for all sleep subgroups, and via anxiety symptoms for all except the Sufficient/Low Quality subgroup (see Table 6). Importantly, between‐subgroup differences in these direct or indirect mediation effects were not significant, suggesting an impact of other important factors.

**TABLE 5:** Direct effect of early ACEs on PRP in the sleep subgroups: Tests of moderation (hypothesis 3).

	1. Sufficient/high quality *n* = 3459 (43.7%)	2. Insufficient/high quality *n* = 2211 (27.9%)	3. Sufficient/low quality *n* = 1048 (13.3%)	4. Insufficient/low quality *n* = 1194 (15.1%)
	Unstandardized/standardized adj. OR [95% CI]	Unstandardized/standardized adj. OR [95% CI]	Unstandardized/standardized adj. OR [95% CI]	Unstandardized/standardized adj. OR [95% CI]
Direct effect of early ACEs on PRP	1.08 [0.92, 1.27], 0.349/1.10 [0.91, 1.32], 0.347	0.97 [0.78, 1.20], 0.781/0.96 [0.72, 1.28], 0.781	1.01 [0.77, 1.33], 0.923/1.02 [0.72, 1.43], 0.923	1.29 [1.03, 1.62], 0.027/1.43 [1.05, 1.95], 0.024
Between group comparisons				
Data are wald statistics (df), *p* value		0.62 (1), 0.430 versus group 1	0.16 (1), 0.687 versus group 1	1.57 (1), 0.210 versus group 1
			0.06 (1), 0.804 versus group 2	3.25 (1), 0.071 versus group 2
				1.84 (1), 0.175 versus group 3

*Note*: Coefficients are exponentiated beta coefficients adjusted for relevant covariates and additional associations.

Abbreviations: ACEs, adverse childhood experiences; CI, confidence interval; OR, odds ratio; PRP, persistent or recurrent pain.

**TABLE 6:** Indirect effects of early ACEs on PRP in the sleep subgroups: Tests of moderated mediation (hypothesis 3).

	1.Sufficient/High quality *n* = 3459 (43.7%)	2.Insufficient/High quality *n* = 2211 (27.9%)	3.Sufficient/Low quality *n* = 1048 (13.3%)	4.Insufficient/Low quality *n* = 1194 (15.1%)
	Unstandardized adj. OR [95% CI]	Unstandardized adj. OR [95% CI]	Unstandardized adj. OR [95% CI]	Unstandardized adj. OR [95% CI]
Hypothesis 3‐via anxiety symptoms				
Indirect effect via interim anxiety symptoms^a^	1.30 [1.15,1.46], <0.001	1.19 [1.07, 1.32], <0.001	1.18 [0.96,1.47], 0.118	1.26 [1.04,1.52], 0.015
Between group comparisons				
Data are exponentiated differences between constrained versus unconstrained unstandardized parameters, [95% CI], *p* value		1.09 [0.93, 1.28], 0.266 versus group 1	1.10 [0.86, 1.40], 0.458 versus group 1	1.03 [0.83, 1.29], 0.784 versus group 1
			1.00 [0.79, 1.27], 0.984 versus group 2	0.94 [0.76, 1.17], 0.590 versus group 2
				0.94 [0.71, 1.25], 0.671 versus group 3
Hypothesis 3‐via depression symptoms				
Indirect effect via interim depression symptoms	1.06 [1.03, 1.08], <0.001	1.03 [1.01, 1.05], <0.001	1.04 [1.01, 1.07], 0.001	1.07 [1.03, 1.11],< 0.001
Between group comparisons				
Data are exponentiated differences between constrained versus unconstrained unstandardized parameters, [95% CI], *p* value		1.03 [1.00, 1.06], 0.068 versus group 1	1.02 [0.98, 1.05], 0.317 versus group 1	0.99 [0.95, 1.03], 0.638 versus group 1
			0.99 [0.96, 1.02], 0.534 versus group 2	0.96 [0.92, 1.00], 0.075 versus group 2
				0.97 [0.93, 1.02], 0.223 versus group 3

*Note*: Coefficients are exponentiated beta coefficients adjusted for relevant covariates and additional associations.

Abbreviations: ACEs, adverse childhood experiences; CI, confidence interval; OR, odds ratio; PRP, persistent or recurrent pain.

^a^ Interim symptoms measured at years 1 and 2.

## DISCUSSION

This study investigated whether sleep deficiency mediates the effect of early and recent ACEs on adolescent pain outcomes, and/or moderates the direct and indirect effects of early ACEs on PRP via anxiety and depression symptoms. Our findings suggest that sleep deficiency may be one of the mechanisms underlying the associations between ACEs and adolescent pain outcomes, but that anxiety and depression symptoms are likely important pathways from ACEs to PRP regardless of sleep status. Notably, our work and that of others highlights the complexity of the relationships among these closely related constructs (i.e., sleep deficiency, mental health and pain symptoms) and causality cannot be inferred from these analyses. Therefore, it remains unknown whether intervening to improve or prevent sleep deficiency and mental health problems impacts youths' risk for developing PRP. However, our findings highlight the potential importance of sleep, anxiety, and depression to future work testing preventative or interventional models.

In this sample, sleep quality and duration partially explained the effect of early ACEs (i.e., ACEs experienced up to age 9–10 years) on PRP. Healthy sleep is integral to adolescent health and functioning (Tarokh et al., 2016), and consistently higher sleep quality may support emotion regulation, cognitive control, and social engagement to the extent that adolescents are better equipped to respond to stressors including pain, even in the context of prior ACE exposures. Indeed, a recent cross‐sectional study of young adults found that aspects of sleep helped to explain the relationship between recalled ACEs and the ability to adapt to adversity (Hillebrant‐Openshaw & Wong, 2023). Thus, better sleep quality across early adolescence may be protective, while sleep insufficiency may exacerbate the risks associated with early ACEs (Fuligni et al., 2021).

Overall, our findings suggest that sleep deficiency may be an important mechanism for the effect of ACEs on PRP during early adolescence (Fuligni et al., 2021). Namely, sleep deficiency may be partially caused by, while also contributing towards, the same mechanisms through which ACEs are hypothesized to increase the risk for PRP (e.g., hyperarousal, emotion dysregulation, inflammation) (Badawy et al., 2019; Nelson et al., 2017; Schønning et al., 2022). Additionally, sleep deficiency and ACE exposure are thought to similarly affect attentional control and negative interpretational biases, which may impact adolescent's perception, evaluation and internalization of painful experiences (Albinni et al., 2023; McMakin & Alfano, 2015; Palmer & Alfano, 2017). Sleep deficiency has also been implicated in the preferential consolidation of frightening or threatening memories, which can exacerbate rumination, pain catastrophizing, and avoidance behaviors, increasing adolescents' risk for PRP (Asmundson et al., 2012; McMakin & Alfano, 2015; Neville et al., 2018). Finally, sleep deficiency is associated with negative mood and social dysfunction, which may hinder the development of close, supportive relationships and other positive childhood experiences which protect against poor health outcomes (Blake et al., 2018; Pugh et al., 2023).

It remains unknown whether preventing or intervening to improve sleep deficiency changes how ACEs impact adolescent pain outcomes. However, limited data suggest that improvements in aspects of sleep deficiency (e.g., insomnia symptoms, duration, quality, or hygiene) are associated with concurrent and sustained improvements in functional disability, health‐related quality of life, mental health symptoms, and frequency of pain symptoms among youth diagnosed with chronic pain and/or mental health comorbidities including anxiety and depression (Law et al., 2018; Logan et al., 2015; Palermo et al., 2017; Åslund et al., 2020). Taken together, findings indicate that targeting sleep problems may be an important component of supporting affected youth. However, these data were primarily derived from small pilot studies (Law et al., 2018; Palermo et al., 2017; Åslund et al., 2020) of youth evaluated in specialized settings (Logan et al., 2015) and diagnosed with clinical sleep disorders (i.e., insomnia), chronic pain, and/or mental health conditions. Further, operationalization of sleep deficiency and approaches towards intervention (e.g., cognitive‐behavioral therapy focused on insomnia alone or in combination with pain; psychoeducation related to sleep hygiene) varied across studies, limiting generalizability. Most importantly, perhaps, no previous studies have examined how addressing sleep deficiency functioned in the context of ACEs/PRP associations. Thus, additional work is required to clarify the nature of these complex associations in youth exposed to ACEs, allowing for greater precision in future prevention and intervention work.

Contrary to our hypotheses, sleep duration and quality did not moderate the direct effect of ACEs on PRP, nor the indirect effects of ACEs on PRP through interim anxiety and depression symptoms. Our findings contrast recent data suggesting that sleep strengthens relationships between ACEs and anxiety among adolescents with chronic pain (Roman‐Juan et al., 2023). It is possible that while sleep deficiency may enhance the effect of ACEs on anxiety and depression, the effects of anxiety or depression on PRP are more strongly influenced by other factors not included in these models (e.g., aspects of the parent/child relationship or parental behaviors). Second, adolescents with higher depression symptoms may present with excessive sleep duration or more time spent in bed (Fuligni et al., 2019). Given that our measure of sleep duration included a response option which overlapped sufficient and excessive duration for youth aged 13–18 years (i.e., 9–11 h) (Bruni et al., 1996; Paruthi et al., 2016), our subgroups may have included participants with higher depression symptoms. It is also noteworthy that the sample's sleep quality scores remained relatively high across years, while rates of sleep insufficiency declined in year three, potentially due to the influence of the Covid‐19 pandemic on school schedules. It is possible, therefore, that the severity and/or chronicity of sleep deficiency in the sample was not adequate to produce moderation effects. Overall, however, findings support potentially important roles for anxiety and depression in the ACEs‐PRP relationship, independent of sleep deficiency.

### Strengths and limitations

Our findings are strengthened by the use of data from the large, diverse ABCD® cohort and the comprehensive cross‐lagged design. Generalizability is limited, however, by the potential for reporting bias, as many ACE items, sleep deficiency, and symptom measures were reported by parents. Parents may be more likely to over‐versus under‐report pediatric sleep problems (Combs et al., 2019; Fatima et al., 2016), leading to a potential underestimation of the magnitude of our coefficients. Similarly, as previously noted, our measure of sufficient sleep duration overlapped with excessive duration for 13–18 year olds. Thus, the sleep subgroups characterized by sufficient duration may have included youth with higher depression symptoms, which may have further reduced effect sizes. While the categorical nature of the SDSC does not allow for the identification of youth with excessive sleep duration, future work may utilize items from the child‐reported Munich Chronotype Questionnaire (Roenneberg et al., 2015) or actigraphy data, which were integrated into the ABCD® protocol in year 2. Indeed, future work may benefit from inclusion of the Munich Chronotype Questionnaire, as chronotype and social jetlag (a measure of sleep disturbances which can be derived from this measure) have been associated with pain incidence in early adolescence as well as ACE exposure (Li et al., 2024; Rojo‐Wissar et al., 2021).

Next, our measure of PRP was derived from parent‐reported CBCL items which may have over‐ or under‐estimated youths' experienced pain. However, external and construct validity of this measure is supported, in part, by the similar prevalence of PRP to recent population estimates (Chambers et al., 2024; King et al., 2011), findings of moderate concordance between parent and child report of somatic symptom presence and frequency (Kröner‐Herwig et al., 2009; Meesters et al., 2003), and higher youth‐reported multiregional pain and past‐month pain‐related interference amongst ABCD participants with higher parent‐reported symptom trajectories (Voepel‐Lewis et al., 2023). Finally, our SEM model accounted for potential bias stemming from the parent‐reported measures of pain frequency, anxiety, and depression symptoms, including correlation coefficients within and across years.

Third, we did not account for post‐traumatic stress symptoms or disorder, which overlap with our constructs of interest but are only assessed every other year in the ABCD® protocol and have a very low prevalence in the dataset (<5%) (Thompson et al., 2022). Finally, the significant direct effects of early and recent ACEs on later pain outcomes suggests additional mechanisms not included in these analyses, which may be elucidated through future research.

## CONCLUSION

These analyses demonstrate that sleep deficiency may underlie part of the effect of early ACEs on adolescent pain outcomes, while anxiety and depression symptoms are likely integral to these associations regardless of sleep status. Continued work in this area may further disentangle the associations among these overlapping constructs and more clearly identify opportunities for prevention and intervention. Our findings reinforce the importance of sleep to adolescent health and functioning and suggest particular relevance for youth who have experienced ACEs and are thus at an increased risk for negative health outcomes including PRP.

## AUTHOR CONTRIBUTIONS

**Thea Senger‐Carpenter:** Conceptualization, data curation, formal analysis, funding acquisition, methodology, writing—original draft. **Anao Zhang:** Formal analysis, methodology, writing—review and editing. **Monica Ordway:** Conceptualization, writing—review and editing. **Sarah A. Stoddard:** Conceptualization, writing—review and editing. **Terri Voepel‐Lewis:** Conceptualization, funding acquisition, methodology, supervision, writing—review and editing.

## CONFLICT OF INTEREST STATEMENT

The authors declare no conflicts of interest.

## ETHICAL CONSIDERATIONS

Informed consent/assent was appropriately obtained from the participants in this study. This study was reviewed and approved by the University of Michigan Institutional Review Board (HUM00225649) and a data use agreement was established with the National Institute of Mental Health (DAR#15008).

## Supporting information

Supporting Information S1

## Data Availability

Data used in the preparation of this article are openly available and were obtained from the Adolescent Brain Cognitive DevelopmentSM (ABCD) Study (https://abcdstudy.org), held in the NIMH Data Archive (NDA). This is a multisite, longitudinal study designed to recruit more than 10,000 children aged 9–10 and follow them over 10 years into early adulthood. The ABCD Study® is supported by the National Institutes of Health and additional federal partners under award numbers U01DA041048, U01DA050989, U01DA051016, U01DA041022, U01DA051018, U01DA051037, U01DA050987, U01DA041174, U01DA041106, U01DA041117, U01DA041028, U01DA041134, U01DA050988, U01DA051039, U01DA041156, U01DA041025, U01DA041120, U01DA051038, U01DA041148, U01DA041093, U01DA041089, U24DA041123, U24DA041147. A full list of supporters is available at https://abcdstudy.org/federal‐partners.html. A listing of participating sites and a complete listing of the study investigators can be found at https://abcdstudy.org/consortium_members/. ABCD consortium investigators designed and implemented the study and/or provided data but did not necessarily participate in the analysis or writing of this report. This manuscript reflects the views of the authors and may not reflect the opinions or views of the NIH or ABCD consortium investigators. The ABCD data repository grows and changes over time. The ABCD data used in this report came from [10.15154/1523041]. DOIs can be found at https://dx.doi.org/10.15154/8873‐zj65.
