# Supplementary material for: The role of sleep deficiency in the relationship between adverse childhood experiences and early adolescent pain outcomes
Source: JCPP Adv. 2025 Mar 23;5(4):e70011. doi: 10.1002/jcv2.70011 (PMC12698282; doi:10.1002/jcv2.70011)

**Supporting Information**

| ACE Item | Survey Question | ABCD assessment and informant* | Years Collected |
| --- | --- | --- | --- |
| Physical abuse | Shot, stabbed, or beaten brutally by a grown up in the home? By a non-family member? | KSADS-5 PTSD Module – Parent | Baseline; Year 2 |
|  | Beaten to the point of having bruises by a grown up in the home? |  |  |
| Emotional abuse | Family member or non-family member threatened to kill the child? | KSADS-5 PTSD Module – Parent | Baseline; Year 2 |
| Sexual abuse | A grown up in the home touched your child in their privates, had your child touch their privates, or did other sexual things to your child? | KSADS-5 PTSD Module – Parent | Baseline; Year 2 |
|  | An adult outside your family touched your child in their privates, had your child touch their privates or did other sexual things to your child? |  |  |
|  | A peer forced your child to do something sexual? |  |  |
| Physical neglect | How often do your parents/guardians know where you are?^a^ | Parental Monitoring Survey –Youth | Baseline; Years 1 through 3 |
|  | If you are at home when your parents or guardians are not, how often do you know how to get in touch with them?^a^ |  |  |
| Emotional neglect | [Primary caregiver] believes in showing his/her love for me^b^ | CRPBI Acceptance Subscale – Youth | Baseline; Years 1 through 3 |
| Parental/family mental health problem | Does either parent have a history of a problem with: depression, anxiety, paranoia, attempted or completed suicide, hospitalization for a mental health issue? | Family History Assessment – Parent | Baseline |
|  | Family member had a mental/emotional problem? | PhenX Life Events Scale –Youth | Years 1 through 3 |
| Parental/family substance use problem | Does either parent have, or have ever had, a problem with alcohol or drug use? | Family History Assessment – Parent | Baseline |
|  | Family member had a drug and/or alcohol problem? | PhenX Life Events Scale – Youth | Years 1 through 3 |
| Parental divorce | Divorced/separated [within the past year]? | Demographics survey – Parent | Baseline; Years 1 through 3 |
| Parent imprisonment | One of the parents/caregivers went to jail? | PhenX Life Events Scale –Youth | Years 1 through 3 |
| Familial financial adversity | Inability to afford: food, telephone service, rent/mortgage, utilities, dental or medical care in the past 12 months; experienced eviction in the past 12 months? | Demographics survey – Parent | Baseline; Years 1 through 3 |
| Interpersonal violence | Witnessed the grownups at home pushing, shoving, or hitting one another? | KSADS-5 PTSD Module – Parent | Baseline; Year 2 |
| Community violence | Saw, heard, or witnessed another person being shot or stabbed? | KSADS-5 PTSD Module – Parent | Baseline; Year 2 |
| Peer victimization | Experienced bullying | KSADS-5 Background Items – Parent | Baseline; Years 1 through 3 |
| Experiencing discrimination | Experienced discrimination due to their race, ethnicity, country of origin, sexuality, gender identity, or weight? | ABCD Youth Discrimination Measure –Youth | Years 1 through 3 |
| KSADS = Kiddie Schedule for Affective Disorders and Schizophrenia; PTSD = post-traumatic stress disorder; CRPBI = Children’s Report of Parental Behavior Inventory | | | |

**Supplemental Table.** Adverse Childhood Experiences (ACEs) in the Adolescent Brain Cognitive Development (ABCD) Study

Note. Early and recent ACE exposures assessed using parent and youth survey responses. Each endorsed ACE was counted as one point.

^a^1 point if youth reported “never/almost never”; ^b^1 point if youth described this behavior as “not like the primary caregiver.”

**Supplemental Figure.** Simplified Model of Hypotheses 1 and 2.


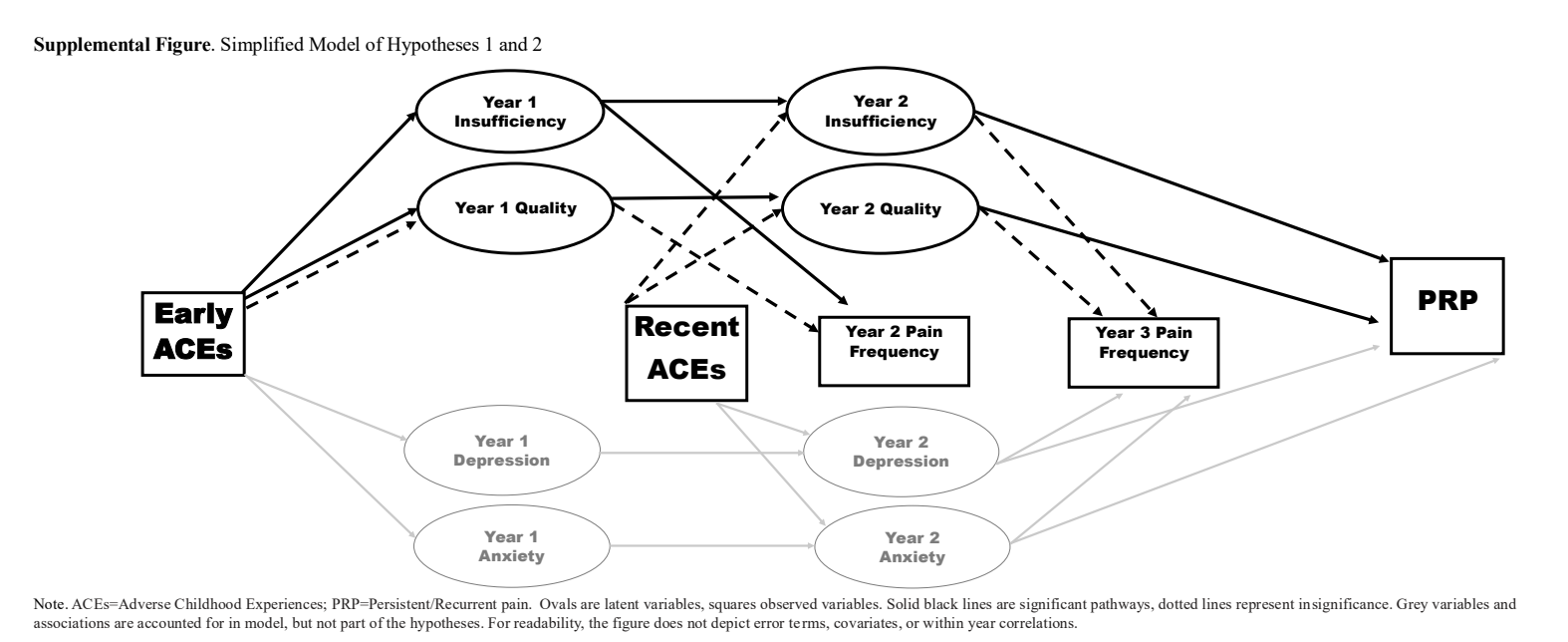

Supplement: Supplementary file 1 — Supporting Information S1 [file JCV2-5-e70011-s001.docx]
